# Researches into the Types of Diseases and Types of Therapy

**Published:** 1858-11

**Authors:** Bennet Dowler


### SELECTIONS.

### *Researches into the Types of Disease and Types of Therapy.* By BENNET DOWLER, M. D.

UNITE *these two methods [the experimantal and the speculative], act like a good physical philosoper, who, in his laboratory,* THINKS AND EXPERIMENTS, EXPERIMENTS AND THINKS, *and makes use at once of his senses and of his reason. Begin with the method* A PRIORI *and give to it by way of counter poise the method* A POSTERIORI. *I consider the identity of these two methods as the only torch by whose light we may find our way.*—[COUSIN'S HIST. PHILOS.

*No experiments can contradict truths derived from the observation of Nature.—The great difficulty under which we labor in our experiments is the immense sacrifice of time and exertion required to imitate the conditions under which we observe the phenomena manifest themselves in Nature.*—LIEBIG.

HAS the type of febrile diseases undergone a fundamental alteration during the last quarter of a century? Was the type formerly inflammatory? Is it now non-inflammatory? Was it sthenic? Is it now asthenic? It is not intended in this place to attempt a demonstration of the positive or negative answer to these questions; but it may be proper to offer presumptive evidence upon this subject—evidence derived not altogether from actual pathology, but chiefly from doctrinal and practical points of view, the validity of which may or may not be questioned.

Yellow fever will, perhaps, serve as well as any other disease for the purpose of. illustration, with occasional glances at other maladies, Has the type of yellow fever changed in New Orleans, to go no further from home? Now, in order to test this question, an appeal may be made to the opinions and practice of physicians, dead and living, during the last twenty or thirty years. Were the physicians of that period agreed in ascribing this malady to localized or general inflammation—to gastro-enteritis or to general sthenic action? Leaving out of view the existing type whether that belong to sthenia or asthenia, it cannot be denied that in New Orleans, at least, physicians of equal respectability and skill were formerly divided in opinion and practice upon these questions. Had this malady been, on the one hand, unequivocally characterized by inflammation, or on the other, by debility and adynamia, would this fundamental antithesis of opinion and practice have existed to any great extent among physicians?

During the period in which many physicians considered yellow fever a gastro-enteritis, sthenic or inflammatory, curable only by repeated blood-lettings carried to fainting, including cuppings and leechings, others treated it either by the expectant method, or by quinine, porter, camphor, opium, and stimulants. Each party claimed superiority as to the successful treatment of the disease by modes really or apparently different. Now it is not reasonable to suppose that all these therapeutic methods were equally good, yet they indicate, at least, that neither was so satisfactory as to be beyond question. A transition state followed. Blood-letting and active purgation declined, as did excessive stimulation, enormous doses of quinine, etc. The resources of Nature; timely, simple, and moderate medication replaced excessive and perturbating remedial agents. Extremists began to approximate, to review their experiences and "reason together."

Did not the type of opinion rather than the fundamental type of disease change? Gradually the rigid antiphlogistic treatment was with few exceptions, abandoned.

There is no necessary connection between the mildness or the malignity of a disease and its fundamental type in respect to its inflammatory or adynamic character. Thus, if yellow fever be an inflammatory disease, it may be, nay, it really is in some cases, mild, or aggravated in degree, though the same in nature or type. Again in puerperal fever (whether that malady be an idiopathic fever, or what is more probable, a puerperal peritonitis) the intensity of the disease in no wise changes its type, though it may modify its therapy. The pure phlegmasiæ exist in every degree without a fundamental change of type.

The aphorism, that extremes meet, is exemplified in therapy, the great finality of medical science. Who could have anticipated that Samuel Thompson, one of the most illiterate men that ever appeared in the medical world, and Professor Bennett, of Edinburgh, one of the most learned, should virtually meet on the same antiphlogistic platform, and agree in denouncing bloodletting even in the most acute phlegmasiæ, and inflammatory fevers? A quarter of a century since, while Broussaism was in the ascendent in the United States, Thompson, who had a million of adherents in this country, maintained "that fever is a friend," and he bound all with a solemn agreement, who practised his patented system, "not to let blood as was common with physicians." He denounced the whole faculty, of whom he said, that they "took the same method to cure a sick man as to kill a beast, that is, bloodletting."

At the present time, Professor Bennett represents a class of practitioners who virtually adopt the same theory in diseases of the most acute and inflammatory class. With them inflammation is, as Thompson would say, a friend—a natural method, to be interfered with neither by blood-letting nor other antiphlogistic procedures. Thompson, however, gave lobelia-emetics to remove what he called the canker from the stomach and bowels. He then raised, to use his own words, as great a heat as he could by steam externally, and by No. 6, or red pepper and alcoholic liquors internally. Some of our modern philosophers feed and stimulate fevers. Where is the difference? Thompson was no skeptic, nor was his emetic weed a mere *placebo.*

A quarter of a century ago, books on liver complaint, and prescriptions for that almost universal malady abounded. A bilious type was assumed for many diseases. Large doses of calomel, powerful, prolonged and repeated purgations characterized the theory and practice, all being based on the antiphlogistic principle, whether as auxiliary to, or independent of, blood-letting. This treatment was carried to an excess for which the epoch was remarkable, and paved the way for that reäction which ever follows unwarranted extremism, be the type of disease what it may.

Admitting provisionally that the type of disease has not changed, it may be asked, how is the existing change in doctrine and practice to be explained and accounted for? In the first place, this change is not altogether fundamental among intelligent practitioners, who limit, without wholly rejecting blood-letting and similar remedial agents, using, yet not abusing them.

The existing restriction imposed on antiphlogistic treatment is due, it may fairly be assumed, to the progress of science. As modern research has been most active, progressive, and successful in almost every branch of the healing art, it would be against all analogy to suppose that therapeutics was either stationary or retrograding all the while, seeing that it is beyond all other branches the most labored, the most important, and has been subject to numerical and other methods, adapted to test and appreciate its claims and achievements. Many unquestioned improvements and successes might be enumerated, even in therapy, affording presumptions at least, in favor of some other points which are still dissented from by individuals.

The great increase of the physical comforts, as to food, clothing, firing, ventilation, lodging, and other hygienic measures of modern times which may be supposed to prevent that deterioration of constitution and general health, which the opposite condition of things entailed a century ago (and much later), when the fevers, especially typhus, were, most probably of an adynamic type, and carried off hundreds of thousands among the poor, the ill-housed, half-clad, and starving.

The invasion of epidemic cholera, doubtlessly tended strongly to convince practitioners that excessive watery purgation, however induced, exhausted the vital forces and speedily ended in death. Here was a vast and all convincing experiment performed by nature, by which millions were struck down. The origin and the progress of choleraïc discharges, though not identical with those produced by active and exhausting cathartics, nevertheless threw a strong light upon their dangerous character, and hence, induced greater caution in their use.

Previous to the invasion of epidemic cholera, the doctrine of Broussais, always adverse to the active cathartic treatment of fevers, had prepared the way, not for the entire adoption of his views, but for a better appreciation of the uses and abuses of cathartic medicines — "Hamilton on Purgatives," did not spare the British alimentary canal, bnt at a later period, the learned Professor Cooke, who wielded a powerful pen, wrote much and was doubtless conscientious, for more than a decennium anterior to the invasion of cholera, operated most extensively upon American intestines with an ounce or even larger doses of calomel mingled plentifully with scammony, jalap and so forth, and drew after him an unexampled number of the faculty, including an almost incredible mass of the population of this Republic, especially in the valley of the Mississippi. Hamilton's purgatives "paled their ineffectual fires" before Cooke's. Cholera and menorrhagia, dysentery, fevers, and innumerable maladies were treated with enormous doses, the history of which will be read with astonishment by the next generation.

Of the many able physicians now living, who adopted Dr. Cooke's views, never fully recognized as valid in New Orleans, there is not probably one who has not repented and reformed, not because the type of morbidity has essentially changed, but because their minds have been changed by the progress of science, by a more enlarged experience, by the logic of events, by a juster interpretation of nature in both health and disease, and by a thorough conviction that, in many instances, neither the cause nor the cure of disease has been ascertained and fixed beyond the possibilty of mistake. No therapeutic generalization has yet been discovered of universal application not even in the acute phlegmasiæ.

The fundamental rule of therapeutics is, *do no harm;* if reason and experience indicate a mode of medication, be it antiphlogistic, stimulant or mixed, adopt and carry it out steadily, cautiously, hopefully, faithfully, unless the logic of events requires alteration.

Bleeding, purgation, blistering mercury, tonics, diet, stimulants, and opiates are not necessarily incompatible. They may by beneficial or detrimental in sthenic or asthenic diseases according to the stages, and individual conditions of the patient. A sthenic affection of the brain or lungs may require blood-letting, purgatives and mercury in one stage, and tonics, stimulants, opiates, and diet in another, all tending to reduce the original diseased action or its ill effects, all counteracting the natural morbidity in the one case, and all coïnciding with the curative action of nature in the other. The *modus operandi* of medicines is not *per se* sufficiently known to enable the practitioner to fix an absolute standard for either sthenia or asthenia without a continuous reference to the carefully observed developments of clinical experience, and the contemporaneous character and import of events, in time, place, condition, season, climate, race, age.

While more or less bias in favor of the present state of medical science, may enter into a comparative estimate with the past, yet the evidence in favor of the superiority of the present, is overwhelming, as might be shown by the simple enumeration of many recent discoveries. Nevertheless, in therapeutics, in which some important discoveries have been made, the number actually available in clinical practice is small compared with the discoveries and progress in physiology, diagnosis, microscopy, physiological and pathological chemistry, pathological anatomy, humoralistic pathology, medical chemistry, pharmacy, and operative surgery. But it may be justly said that the efficacy and the inefficacy of medicinal agents, the danger of entire non-medication, and of excessive medication, the destructive and healing powers of nature, are now better appreciated than formerly. The excessive confidence once reposed in very active medication is lessoned, or replaced with extreme caution, and, on many occasions, with reserve and timidity, the pretensions of art being resigned in favor of Nature, the *vis medicatrix naturæ*, of which more will appear in the sequel.

Upon reviewing the whole ground, the following conclusions may be regarded as probable: The sthenic type of disease during the first quarter of the present century and at the commencement of the second, was not so general nor so strongly marked as to justify the excessive blood-letting, purging, and other antiphlogistic measures which were generally practised. The non-medicationists of that period and of the present erred, and still err, in adopting the opposite extreme. The great body of enlightened physicians now occupy a middle ground between these extremists, inclining, perhaps, to the opinion that diseases are less inflammatory or sthenic than formerly, without adopting the opinion that all diseases are asthenic or adynamic, the antiphlogistic as well as the supporting treatment being adapted to individual cases. The former, however, is rarely, if ever, required to the exaggerated extent practised from twenty to thirty years ago.

But it has been, and is still asserted by some of the most prominent medical teachers and authors of the present day, that the type of disease has undergone no change, and that the decay of the antiphlogistic treatment is due to the progress of science. Thus, if yellow fever always was, and still is a sthenic malady for the cure of 'which blood-letting must be carried to fainting' and be repeated until the malady shall yield or terminate in death, how shall the general disuse of this remedial measure at present be accounted for? If that faith exists, its works have nearly disappeared. If, on the other hand, the faculty have laid aside this treatment because they have found it ineffectual or injurious, the disease in the mean time having been of the same type, how is this to be explained without making a candid confession of previous ignorance, incompetency, and mal-treatment? Is mankind addicted to bearing testimony against itself?

Now, admitting what history teaches, namely, the weakness of human nature, a readiness to excuse one's self for errors and faults, (the first man excused himself by blaming his wife,) nothing could be more plausible than to accuse the yellow fever of 1837 and '39, and especially of 1841-2-3, with sthenia, and that of 1853 with asthenia, seeing antiphlogistics would suit many physicians in the one case, and tonics, etc., in the other. Without making such a charge against any one, it may be here said in all charity, that a temptation to adopt such a mode of exoneration rather than the confession of error, incompetency, and mal-practice, exists. Hence the wisdom of the prayer—"Lead us not into temptation."

The faculty of New Orleans who have witnessed the epidemic yellow fever of 1837, and every subsequent invasion up to the present time, have had beyond all others opportunities for investigating and determining the type of that disease, and the comparative efficacy of the various methods of its treatment. The recorded experiences previous to 1833 and '37, not excepting a few reports of the Physico-Medical Society, are meagre and unsatisfactory. The memories of preceding epidemics, dim and ever fading among the most retentive, are now no longer available, being extinguished by death.

The faculty, who for the last twenty years have battled with New Orleans epidemics, represent many nationalities, have a medical clinic of various nations and races, and have practised the different methods of treatment which had been previously recommended, or such as appeared to promise more favorable results—whether the simple ptisans of mulattresses, or, active mercurial cathartics-rigid non-medication, or twenty to sixty grain doses of quinine—porter, or blood-lettings, limited only by swooning, followed by cupping, leeching—forced perspirations, in closed rooms, under blankets or cold spongings, iced drinks, and free ventilation with open windows, the lightest possible coverings—strcyhnine, or sweet spirits of nitre—warm tea or cold hydropathy—a strong infusion of coffee, or ten grains of tartar emetic hourly, etc.

These and many other remedial measures, whether viewed as specifics, or as adapted to remove special symptoms and complications, were put in requisition either by the faculty, or by non-professional persons, charitable associations, mulattresses,* etc. and cannot fail

---

* The following letter which appeared in a Northern Journal during the epidemic of 1841, is not altogether a caricature:

THE YELLOW FEVER.—The yellow fever, although it has visited us twenty times in the last thirty years, is by no means treated in a uniform way by the doctors. One bleeds—another says to bleed is to kill, a third gives a decoction of green coffee, a fourth calomel; a fifth quinine; a sixth the Sydenham portion; a seventh purges with salts; an eighth laughs at all purgatives, and claps blisters on the feet and stomach; a ninth covers you with blankets; a tenth congeals you in ice; and some do nothing at all but nurse the sick man, letting nature take her own course. Of all these treatments, it is hard to say which is the most *un*successful. What kills one, cures another; and the work of the destroying angel goes on regularly and proportionate, and, as if, by way of satire, the malady has fallen upon the doctors themselves. Two promising young physicians, Lafon and Delavigne, died a few days ago of the epidemic. The latter belonged to the American doctors; they bled him, etc., etc., following what is called the American practice, every effort was used to save him. When he died, the French physicians shrugged up their shoulders and sneered at their American brethern; but down went Lafon, and in spite of "sharp-pointed soup," or *bouillon pointu* as they call a certain mode of giving a cool dose to the bowels, in spite of blisters and lemonade, etc., etc., off he went, to the utter confusion of the French practice. With this total want of success on all sides, you see what must be the dreadful state of anxiety, fright and uncertainty in which those who are unacclimated are constantly kept.—Strange, it seems to me, that after so many years of experiment, and of experience, nothing definite or settled is arrived at in the treatment of this inferto afford the philosophical observer data for serious reflection, and some probable conclusions.

The faculty, always the most competent judges, have been at length very generally convinced from abundant experience, that in not a few cases the fatal tendency of yellow fever cannot be averted by any known remedy. They look with distrust on all modes of treatment which claim to be universally successful.

Distrusting polypharmacy and pertubating remedies carried to extremes—it may be repeated, they find in nearly all modes of treatment valuable resources which may be relied on in particular cases, either as curing, or as enhancing the chances of recovery when skillfully applied.

The apparent antagonism of types of disease, and of modes of treatment, is, after all, for practical purposes, to a great degree, conditional and contingent. Thus, as already stated, an acute pneumonia, pleurisy, or peritonitis which may require blood-letting in the early stage, may afterwards equally require stimulants and tonics to obviate debility, and simultaneously opiates and mercury, to remove exudations, effusions, indurations, or other effects of the antecedent stage.

Although it is not intended to extend this paper by data copiously recorded in manuscript, illustrative of the progress of medication in the yellow fever of New Orleans, the following note made a few years ago will show the decline in regard to heroic doses:

During the prevalence of the yellow fever *anno* **** I copied from the books of the Hoprital, the prescriptions in six wards from September 1st, to the 13th inclusive. I omitted simple drinks as ice-water and lemonade. I estimated the average daily number of yellow fever patients at sixty, and other patients at thirty. In thirteen days these patients used, of calomel, twenty-five grains; blue mass, ninety-six grains; salts, one dose; castor oil, four doses; enemata, twenty-eight; blisters, nine; general blood-letting, four, and local, three; cold to the head, one. In one ward sulphate of morphia was given oftener than any other medicine, but the number of doses is not stated. Hence, in six wards the daily consumption of calomel was less than one grain.

In another large ward filled chiefly with yellow fever patients, the

---

nal sickness. As to myself, I fear it not. I was born yellow fever proof; for I write from my native city.

It is now settled that yellow fever is not caused by miasmatic exhalations, nor by excessive heat, for it is extrmely fatal during a season of daily rains, which constantly cool and purify the atmosphere. A fresh north wind is now blowing. What is the cause of the epidemic no doctor can tell.

prescriptions for one day comprehended, one porteree; six ice-water; four gum-water iced and lemonade; two blisters; one sinapism; six ounces brown mixture; two grains of tart. emet, and half a drachm of syrup of morphia. In another similar ward, in the same month, the prescriptions for two days may be thus summed up; foot baths, two; barley water with elixir of vitriol, four; quinine juleps, four; enemata, three; cold to the head, one; quinine, ten grains; camphor lineament, one; blisters, two; castor oil three; lemonade six; gum-water 6. In some-other wards, the swooning-blood-lettings (*les saignées syncopales*) were practised as fully as if M. Bouillaud had been the medical attendant. Many physicians, including M. Thomas, considered the epidemic of this year, inflammatory, requiring the long continuance of and energetic antiphlogistic treatment. *Traité Prat. de la Fièvre Jaune. Paris.*)

Several medical men (during this epidemic, who practised repeated syncopal blood-lettings ("*saignées syncopales réitérées*") lost only four in the hundred, others one in fifteen, according to *their* published statements!

A writer on the yellow fever of New Orleans, opposed to cathartics in this disease, who "never saw an instance fit for tonics, nor hæmorrhages except when mercury had been taken," and who, by bleeding, "in the horizontal positon," was enabled to give his statistics in the following pleasing words—"out of my seventy-five cases only six died." Nevertheless, Dr. La Roche in his learned and colossal work on yellow fever, finds that in the United States, the mortality statistics of this disease give nearly one death to every three patients, instead of one in twenty-five, one in fifteen, or one in twelve.

Another medical gentleman during a later epidemic, reported that he had seventy-seven cases, and cured all but three—another that he had treated one hundred, and lost four. A respectable apothecary, however, (since dead), declared that he knew of many, including one entire family, that died under the treatment of this gentleman at the same time and in this same epidemic. This treatment began now to assume a new name, that of "*spoliative* blood-letting," instead of *jugule.* Of this latter, the late M. Magendie, in his published lectures, hintəd that it is not the disease, but the patient that is *jugulé.*

The late Dr. Drake, in summing up yellow fever statistics, found that for sixteen years ending in 1843, the mortality in the Charity Hospital's "accurate registry" amounted "*to one out of two*," and that "all the available data give an average mortality over forty per cent." Private practice, is for obvious reasons far more successful.

These brilliant cures were not witnessed in the public hospitals; and instead of dwelling upon them, it may be better to return to the line of investigation with which this paper set out.

It is not Thompson, but certain eccentric skeptics among the otherwise learned, who contend that inflammations and fevers cannot be in any instance cut short or arrested; that these maladies must run a definite course uncontrolled, as regularly as the seasons, without hindrance from art, as if nature is altogether competent to practise physic, but is the worst of quacks in setting a broken bone, reducing a luxation, extracting a stone, or a tooth, reducing a hernia, closing a hair lip, and in performing cæsarian and most of other curative operations. That nature's morbid processes are essentially healthful and curative,, and that the healing art can do nothing but aid, abet and accelerate the march of the former, are assumptions too absurd to be admitted, being not only contrary to experience and analogy, but self-contradictory, claiming, yet in no wise receiving unequivocal support from recent microscopic investigations. The latter how important so ever they may ultimately prove in pathology, are not sufficiently matured for a basis of therapy; though they may be admitted without invalidating the question of sthenic or asthenic treatment.— Nor can it be justly affirmed that the great advances recently made in diagnosis prove or disprove the possibility of controlling inflammations, fevers, etc. Diagnosis, though of the utmost importance, does not necessarily carry with it the method of cure in phthisis, cancer, cholera, heart-disease, etc.

The diagnosis of cholera is easy. But it does not follow that the cure can be deduced from this diagnostic knowledge. The precision with which consumption is now diagnosticated is among the most remarkable advances in modern pathology, but the mortality statistics of the present time will show that the cure of this disease has not progressed in a ratio comparable with that of its exact diagnosis. Hence the fallacy of theorizing in therapeutics solely on this basis.— Intermittent always existed, and is easily diagnosticated, but this latter did not contribute an iota towards the discovery of the Peruvain bark.

This statistical argument, however decisive in itself, is virtually neutralized by the significant fact that every mode of treatment from the heroic to the infinitesimal, claims its protection and takes shelter under its autocratic sanction. Figures cannot lie. In the celebrated medical school of Edinburg, Dr. Henderson, Professor of General Pathology, finds figures proving the superiority of his homœopathic treatment, and in the same school, Dr. Bennett, Professor of the Institutes of Medicine, teaches that his figures in favor of no treatment, at least no antiphlogistic treatment, are still more favorable, and that the figures in even pneumonia for instance, "*satisfy him that blood-letting lengthens rather than shortens the progress of the case. The real disorder is prolonged, and rendered proportioually more fatal by that practice.*" Now M. Bouillaud has tables showing that in numerous cases of French pneumonia, in which blood-letting averages for each patient four pounds and nine or ten ounces (French), yields with great promptness, giving the minimum of mortality, when the disease is thus *jugulated.* In some hard cases, ten pounds were required to jugulate or strangle this disease. (*Philos. Méd.*) This is nearly as much as I have known to be taken in individual cases of yellow fever in New Orleans. Prof. Bouillaud's practice was fully tried, and even his neologies adopted, in this latitude, and that too, by able physicians, a number of whom, the first surgeons of the world must own as their equals, when put to the test. This subject will be referred to again.

But to return to the Edinburgh Professor:

Dr. Bennett's test disease, pneumonia, is a fair type of inflammation as it exists in the parenchymatous viscera. Here he plants his theory, appealing to statistics to show the pernicious effects of antiphlogistic treatment and the truth of his views. In order to accept his therapy of the phlegmasiæ, it is necessary to discredit the almost unanimous testimony of the past and the present. The presumption is, therefore, altogether adverse to his isolated opinion.— But this is not all. His medical compatriots, and more especially Dr. Bell, deny the authenticity of his special statistics. In this state of the controversy and uncertainty, the preponderating evidence is by no means in his favor. A small portion of Dr. Bell's paper invalidating Dr. Bennett's statistics and logic has already appeared in the New Orleans Med. and Surg. J.; its length prevented its insertion entire.

Hippocrates after enumerating the symptoms of acute pleurisy and peripneumonia, lays down this rule of treatment, namely, "venesection; the quantity of blood drawn to be proportioned to the constitution, the season, the age and the color of the patient; if the pain be acute, the bleeding should be boldy pushed to syncope; afterward, an injection is to be administered." Has modern microscopy led to a sounder rule? Experience has limited its application by the circumstances indicated by Hippocrates; the syncope is an accident, but not the object aimed at, which is relief. Centuries later, Celsus said with due circumspection that for this disease, the vigorous should be bled—the feeble cupped: (*Oportet, si satis validæ vires sunt, sanguinem mittere: si minores, cucurbitulas, etc.*) (l.iv.c.vii.)

M. Bouillaud lays great stress upon the diagnosis of disease as the certain key to the cure: "If there be an axiom in Medicine," says M. Bouillaud, "it is, that no disease exists without a seat. The determination of the seat of diseases is one of the most beautiful conquests of modern medicine." *Essay on Med. Philos.* 259.) This determination of a special and invariable seat of yellow fever remains to be revealed. Early in this century some of the most prominent British writers, for instance, Dr James Johnson, considered the seat of yellow fever to be in the liver, the disease being virtually a hepatitis, while in Paris it was called a gastritis. Now, assuming provisionally, yellow fever to be a local inflammation of the stomach and bowels, or of the liver, its mode of cure, blood-lettings pushed to syncope is not a necessary consequence of this pathological condition, nor is the absolute interdiction of purgatives theory proven to be necessary, as the expulsions of fæcal and morbid excretions, not to mention their antiphlogistic actions in other respects, may be beneficial. Experience alone is competent to decide in these cases. Analogy, nay, direct evidence, shows that a treatment whieh reduces the strength and vital forces without diminishing the disease, is injurious.

On the supposition that blood-letting carried to syncope cuts short, or controls the violence of fevers and inflammations, there is great danger in its continued repetition, seeing that it may prostrate the inherent vital forces, without which the natural cure, so to speak, must be precarious, for without a certain *quantum* of this inherent vital power, tonics, stimulants and diet, must fail to insure recovery how much so ever, the original disease or its accidental complications may be subbued.

Nevertheless, the natural method of curing a malady is in many cases wholly unreliable, unless art should interfere to remove or check injurious agents, morbid conditions, and unfavorable circumstances so that Nature may be the better able to cure the disease by recovering her normal reign, abdicating always in favor of old age.—Therapy is virtually based on a quality, namely, morbidity and health — a duel in which the doctor may often heal wounds not necessarily mortal.

A philosopher after much research, devoutly concluded it was a remarkable proof of the goodness of Providence that great rivers always ran by great towns! The Pantheistic notion that the whole universe, nature, matter, disease, etc., is God, may be distrusted in clinical medcine. Nature in disease is very often an arrant quack of the most dangerous kind, a blind, unintelligent necessity, without medical education, to be opposed with force arms. The founders of christianity performed miracles to oppose Nature in disease. Doctors of these latter times not being thus endowed, fight or control her, not the Almighty, with physic, scalpels, splints, forceps, chloroform, vaccination, and so forth.

Broussais' doctrine, which was almost exclusively of the inflammatory type, had scarcely begun to wane in New Orleans, when the work of the distinguished Professor Bouillaud, on Medical Philosophy appeared. (*Essai sur la Philosphie Médicale et sur les Généralités de la Clinique Médicale.* Paris. 1836.) His type of disease was also inflammatory and his remedial type (which, it is said, he still adheres to) was blood-letting carried to an extreme without parallel. The English language is too poor to express his sanguinary practice: Swooning and fainting are innocent babes compared to his *coup sur coup*, *toute puissante*, *jugler*, etc. The result of this *Thugging practice,* which he calls the new method, are given in his book, fortified with a profusion of arithmetical tables to establish its almost uniform success in pneumonia, pleurisy, heart inflammations, inflammations of the stomach, bowels and other abdominal organs; also in rheumatism anginose affections, erysipelas,* and fevers.

In typhoid fever, M. B. claims to have cured all but twenty-two out of one hundred and sixty-eight cases, nearly one in eight, instead of only one in three according to MM. Chomel and Louis' statistics. These M. B. calls "the results of his method—the new formula." Be this as it may, the treatment of yellow fever in New Orleans by

---

*From April, 1834 to March, 1836, M. B. treated fifty-three cases of erysipelas of the face, having cured all with astonishing facility and rapidity by local blood-letting, employed *coup sur coup*, (p. 415,) amounting in several cases to four or five pounds;—"*la malalie etait reellememt jugulee.*"

Van Helmont, (born 1577 died 1644) who rejected blood-letting which he maintained exhausted the *archæus* or vital spirit lost his life from an acute pleurisy, which, as Guy Patin, a comtemporary doctor, said, might have been saved by venesection. This celebrated Guy Patin practised "spoliative blood-letting" equal to Rush, if not Bouillaud himself, having bled his son, in a fever, twenty times—the king's physician, sixty-four times for rheumatism.—"There is not," says he, "a woman in Paris who does not believe in its efficacy even for her infant when affected with small pox, measles, convulsions, teething," etc.

serial sanguineous emissions limited only by swooning—*coup sur coup juguler*, ended in more than distrust, notwithstanding the ability, skill, zeal and benevolence of its advocates; some of these are now dead, others have returned to their native skies, and many it is believed, after an abundant experience and matured reflection, have modified or dissented from their previous opinions.

"I was," said Bordeu, "a dogmatist at twenty; an observer at thirty, an empiric at forty, and now at fifty, I no longer have any system," When I was young, said a physician, I had fifty remedies for every disease; now I find fifty diseases for which I have no remedy. True; most venerable centenarian! But have you not seen veritable discoveries, almost innumerable, particulary in surgery? Did vaccination, quinine iodine, anæsthetics, and other modern achievements enter in your most youthful anticipations? Nevertheless an enlightened experience tends to reduce or rather to exclude from practice a vast majority of the drugs enumerated in the dispensatory.

The diagnostic question of sthenia and asthenia (now that diagnosis is viewed as the *io triumphe* of modern medicine) would seem to be one upon which diversity of opinion should no longer exist; but in practice it is not so, judging by the actualities of therapeutic standards. It will be seen in this paper that many physicians of equal ability in New Orleans, (where yellow fever is probably best understood) have in every epidemic for a quarter of a century, differed in opinion and therapy on this question. It is the same in other cities. For example, in Paris, two of the most eminent medical men, the best representatives of these two fundamental types of therapy, M. Bouillaud (whose extreme antiphlogistic treatment has been already adverted to) and the late M. Magendie, who adopted the most opposite practice, will serve to prove the truth of this proposition.

A few years ago M. Magendie, in his lectures in the College of France, summed up his conclusions as follows:

He said, while disavowing all belief in homœopathy, that "a physician would cure a patient with globules, if the patient had faith in them, better than with the most appropriate medicines, if he distrusted their action. The benefit of blood-letting is due to the imagination or moral effect. For more than ten years I have not found it necessary to have recourse to a copious bleeding; in other words, I have endeavored to act on the mind of the patient rather than on the circulation." In the *Hôtel Dieu*, M. Magendie treated pneumonia and other acute diseases with hygienic means, as temperature, diet, etc. Here then are two among the greatest teachers, writers, diagno. sticians, practitioners, and experimenters of the school of Paris, who present therapeutic, if not diagnostic antagonism unparalleled among eminent men in New Orleans. The rule which some have proposed, that is, trial blood-letting as a test of type for sthenia or asthenia is fallacious. I have witnessed cases wherein I could not discover any of the usual phenomena of inflammatory action, in which six or seven pounds of blood had been taken in from two to three days without a fatal result.*

As the recent counter-march from the antiphlogistic camp to that of asthenia and semi-medication is due neither to the British nor American schools, but to that of Paris, or rather that of a party in this capital of which M. Magendie was autocrat (whose funeral eulogium has recently been pronounced), it may not be irrelevant to the scope of this paper to recall to mind some of his fundamental views, which will be condensed from his work *on the blood.* Whether these views be true or false the reader will determine for himself. Truth cannot be crushed out by the great name of Magendie, if he shall be found in error.

"When I commenced my medical career, imbued with the prejudices of the schools, like my brethren I paid my tribute to scholastic dogmatism; that is, I believed in inflammation, irritation etc., as so many articles of faith." 107. In repeating some experiments of Sir B. Brodie on the ligation of the common bile duct in which all the animals died of peritonitis, M. attempted by copious blood-letting before experimentation to prevent this, but found that it had the contrary effect, increasing the inflammation, etc., which also, induced him to condemn bleeding before surgical operations. 107-8. He declares that by bleeding animals (dogs) from time to time he finally kills them with inflammation, produced by the blood-lettings, as engorgement, œdema, pneumonia, and, "the entire train of what people call inflammatory phenomena." The serosity of the blood increases, fibrin diminishing in these cases. 19. "What on earth is the meaning of irritation? What sense is there in applying the word inflammation to our organs? Do our tissues really take fire? I confess I know of no example of such a phenomeuon. 32. The fundamental point in the theory of the blood is, that in order to suppoıt life it must be coagulable; if it loses that property existence is threatened.

* In 1809, Dr. Lucas, of Virginia, bled Capt. Niblett, in less than a month 57 pounds troy, besides cupping, leechings and the use of setons, for a pneumonia.—*Med. Repos.*

This is what occurs in the greater number of epidemics. 28. Viscosity of the blood is necessary to its free and healthful circulation.—Thin blood or water will not enter where viscous will. 35. Blood-letting changes the relative proportion of serum and clot, changing the quality of each, with a consequent change in the organs causing the very disease which venesection is intended to cure. Uncoagulable blood cannot traverse the capillary vessels. 40-1. He refers to "rheumatism which yields to bleeding, tartar emetic, to every imaginable kind of treatment—yields above all to rest, and diluents. In my hospital practice I never have recourse to the lancet, to tartar emetic or to leeches, yet all I have treated have recovered." 177. He opposes tartar emetic in thoracic affections as given by Laënnec' maintaining that it attacks the blood chemically, decomposing some of its elements, producing in the lungs the diseases which it is given to cure.

"In serous apoplexy, acute hydrocephalus, etc., venesection diminishes the proportion of fibrin, of coloring matter of the blood, increases that of serosity, facilitates exhalation, and therefore, seems to me a most likely plan to aggravate the violence of the symptoms." 110.

"I hesitate not to declare, no matter how sorely I shall wound our vanity thereby, that so great is our ignorance of the real nature of the physiological disorders, called diseases, that it would, perhaps, be better to do nothing, and resign the complaints we are called on to treat, to the rseources of nature, than to act, as we are to often compelled to do, without knowing the why or wherefore of our conduct, and at the obvious risk of hastening the end of our patient." 88.

Amid the difficulties and doubts which still reign in pathology and therapeutics, the inquirer should not adopt as ultimate truths the prevailing illusory phrases, "*vis medicatrix natræ*," "God in disease," the natural method," "expectant method," "*les juguler*," "self-limited diseases," etc.; instead of these, let him substitute farther experiments, observations, and deduction. Thankful for what science has already achieved, let him labor, hope, wait.

If the epidemics of New Orleans have taught its medical faculty that no medication yet known is beneficial, this is a discovery of high import to the well being of society, since it will soon or late obtain influence over lay-medication, the type of which is very active, consisting of emetics, purgatives, etc., and consequently, injurious. If the former modes of treatment have been too active and perturbating, now is the time to reform them—to restrain the interference of art so as not to thwart nature; if nature can be assisted with physic and hygienics, be it so; prescribe the formula. This antithesis between nature and art in therapy, is as fanciful as it would be to assume an opposition in agriculture, manufactures, cooking, etc, Intelligence and skill harmonize these two agencies and give mutual support.

If sheltering themselves under the authority of Sir John Forbes, physician to the Queen, Professor Bennett, the late M. Magendie and other physicians still more skeptical in the art of healing, and altogether opposed to medication, the faculty should renounce the practice, but not the study of medicine. much evil and probably some good would be the result. History, daily experience, the hopes and fears of mankind prove that remedies, good or bad, will continue to be given for the removal of suffering and for averting the danger of impending death. As almost every individual would have a different theory, method, remedy, or dose, this universal empiricism would afford the true physician new data for study. Some of the most remarkable cures I have witnessed, have been accidentally made by persons ignorant of physic.

If the signs of the times be not illusory, medical opinion and practice are receding from the reliable land-marks of the past. The antithesis of the healing power of Nature and Art, a *petitio principii* at the best, is gaining adherents and assuming consistency under various disguises and scientific presentions warranted neither by experience nor medical statistics. Thus, Prof. Bennett teaches his class the fundamental doctrine "*not to attempt to cut short even pneumonia,*" etc. Some medical men who advocate a positive therapy, qualify their support with so much reserve and lurking skepticism as to place themselves under a neutral flag, or among friendly enemies more dangerous than the openly hostile homœopathists, hydropathists, spiritualists, and self-styled reformers of the physico-medicalist and Thompsonian sects.*

Legitimate Medicine has in all ages encountered assaults by enemies from without and by dissension from within its pale, but at no

---

* An epitaph on the tomb of the old Ambrose Parè, now much quoted and true enough in a certain pious sense, reads thus: "*Je les pansey et dieu les quarit.*" "Empirics may boast that they cure, and doctors of divinity may sustain them, but the physician knows that it is God who healeth all our diseases." Whereupon *The Southern Medical Reformer* (Thompsonian) for July says: "Such a letting down! What? The Doctors cured no diseases!—What then, are they good for? If they cure none, don't they sometimes make them worse? Have the thousands of constitutions that the Doctors have ruined confessedly, been sacrificed to a vagary of the imagination?"—Since the age of miracles has passed away, "God healeth our diseases," as he maketh our cotton, sugar, railroads, steamboats, telegraphs; or cutteth the stone out of the bladder, etc.

time have adverse circumstances or illusory opinions been more prominent during the last fifty years than at present. notwithstanding the scientific progress made in all this period. During the last few months the changes of opinion originating within the profession have been greater perhaps than ever occurred in so short a period—changes the most disastrous or fortunate—the most disloyal and false, or disinterested, truthful and beneficial to society. If medical treatment be a snare, a cheat, and mischeivous; if nature be competent to cure without the assistance of art, and further, if art, how skillful so ever may its interferences, tends only to enhance the dangers of the sick, serving but to prolong their maladies, the physician's "occupation's gone." Medical colleges, journals, and books should sing hosannas to morbid Nature, and hurl maledictions against her enemies and the enemies of the afflicted, that is, the doctors.

An author who has unfortunately written a book, or essay in which he has "made up and expressed an opinion" upon a method of treatment, is apt to be biased in its favor. "When Dr. Sangrado was told that all his patients died, and that he must, therefore, alter his system of bleeding and warm water, he replied—'Impossible? for I have written a book upon it.'"

"O, that mine enemy would write a book!" said Job. If he meant a book on the practice of medicine, written to sustain an ill-founded and exclusive method of treatment, this prayer impends no good but great danger to the enemy aforesaid, as he exposes himself without cover to the fire of all who differ from him, and worse yet, soon or late he may fear annihilation from the batteries of experimental research, or from some newer dogma equally unfounded.

It was said of Hamilton's book on Purgatives, that it quadrupled the sale of purgatives in England. When it was translated into French' more than thirty years ago, M. Tavernier in his review of it, exclaims, "Ah? if French physicians could adopt the practice of Dr. Hamilton, what a number of people could consider it as the regeneration of medicine in this country? With what exultation will the whole class of pharmacopœists, view the termination of a calamitous revolution, by which their shops have been left deserted and their drugs allowed to rot? If purgatives should come into vogue, what will become of the poor leeches? Americans, however, will not allow apothecaries to starve for the glory of a theory. If the faculty renounce drugs and adopt the so-called "natural method," the people and the apothecaries will not. They will take physic as well as vote, and quack advertisements which already occupy a large space in the newspapers, will increase in proportion to the decline of prescriptions by the faculty. Abstractions die, but druggism is immortal.

It does not follow because neither pneumonia nor yellow fever, etc., can be *thugged* by "spoliative blod-letting," nor by "the abortive treatment," that treatment can in no wise directly contribute to the arrest or the favorable modification of the disease. Suppose, for example, that you ride twenty miles among the hills of Western Virginia, to see a robust farmer suffering from inflammation of the luugs, or pleura, and after bleeding him, you hear him say, the pain is gone and then, perhaps you see him turn over and go into a sound sleep.— In a few days after he gets well, with or without other treatment. Are you to conclude with some closeted microscopist whose facts and theories concerning the cells, their anatomy, physiology, combinations, growth, life, and decline are truly interesting, yet do not afford a basis for medication, nor prove the expediency of doing or not doing any thing whereby "*the exudation of the liquor sauguinis*" in inflammation may be modified—I say, are you to conclude, that you have in the malady aforesaid been guilty of mal-practice in furnishing aid and comfort to the enemy? Are you to adopt a histological *non sequitur* instead of falling back on clinical experience and medical statistics?

Mother-wit is a guarantee that the unnatural defection in the ranks of Medicine, now prevalent, can only be of temporary duration. Whatever changes may occur in the type of diseases, the very skeptics whom "too much learning hath made mad," will consent to be purged, blistered and bled, or stimulated in the trying hour, just like their more loyal brethren who still practise physic under the belief that it is an honest business, and withal beneficial in removing obstructions or morbid conditions, so that the healing powers of nature may the more readily rehabilitate the healthful functions of the economy.

These questions of type and of therapy, rather suggestions in relation to medical facts and the opinions deducible from them, including the modes of treatment found to be beneficial or otherwise, are submitted to readers, who are earnestly resquested to investigate these subjects and give their results to the world in this Journal. Discussion may elicit the truth, and if men cannot be convinced and converted, they may be induced to think and to reason. "I question," says Mr. Surgeon Johnston, the author of *Nuces Philosophiæ*, "whether any man was ever convinced by the author of another. All that a reasoner can do is to set his readers *a-thinking* in the right direction. He gives them a clue, and then they either dress up his arguments in their own language, and please themselves with believing them their *own*, or else they do really discover new arguments of their own, on the same side by which they are convinced. And there is reason for this. For the same natural law which makes it offensive to be beaten with a stick, makes it offensive to be beaten with an argument. There is a distinction but no difference."

Home facts and home opinions are needed. What has been done what is now doing abroad, should not be overlooked or neglected.—But the rural practitioner in the South will not always find in the clinical reports from foreign lands, from crowded pauper hospitals, under different skies, among physically deteriorated and crowded populations, precise and available information to guide him in the treatment of patients of different climates and races, scattered over a territory as large, perhaps, as some of the German States. A home practice or a home book is much needed—a periodical book of record.—Such the readers can make this Journal. A faithful history of medical practice contributed to its pages from all parts of the expanded South, would be of mutual advantage for mutual instruction, and would greatly advance the art of healing, especially in rural localities, to which the practice in the filthy purlieus of crowded towns and hospitals, may not be always strictly applicable. Such contributions, though small and sometimes imperfect, viewed singly, become, in the aggregate, of inestimable value, and would soon rival in utility, if not in splendor, more pretentious volumes. Home essays, imbued with home practice and thoughts, adapted to home populations and places, are due to ourselves, and will command the respect of non-resident medical compatriots and foreign cultivators of science. An American school! Yes, a Southern school with its four millions of negroes, and peculiar climate, diseases, and topography, is a desideratum.

Is there not a necessity for inquiring whether there be not a rural as well as an urban school of clinical medicine? The city physician, usually the bookmaker or monographists, sees in hospitals and among the poor who greatly preponderate over the affluent, a comparatively debilitated population, badly housed, badly nourished, badly aërated, and often badly intoxicated with bad liquor? Is the city type of therapy adapted to the vigorous population, white and black, in the country, where agricultural pursuits constitute the employment of nearly all, where food is fresh, wholesome and abundant, where the muscular forces are highly developed, where the blood is aërated with pure breezes from plain and hill, and where, it may be presumed, an acute malady treated with more or less energy, will not be followed by a collapse of the vital forces? Let the country physicians teach, that is, write, as well as the physicians of the overgrown cities of Paris, London, New York, Philadelphia, New Orleans.

The tendency of medical opinion in favor of the importance of hospitals as schools of clinical education is great, increasing, and, in this point of view, ought not to be diminished. But there are so many unfavorable mental influences and hygienic circumstances which modify hospital practice, that the student who accepts as model types of disease and therapy what he observes in such institutions, may underrate the prevalence of sthenic maladies and the efficacy of antiphlogistic measures. Under the best practicable, mental, hygienic, medical, surgical, and obstetrical treatment, where from five hundred to one thousand sick persons are crowded into one building situated among filthy streets, the chance of being cured is small compared with private practice in urban, and still more in rural situations. Should the popular mind ever arrive at a thorough appreciation of this fact, a revolution will probably take place in regard to the increase of large charity hospitals, notwithstanding their advantages as schools of instruction, and their economy in diminishing expenses. A growing belief, whether ill-founded or not, that most diseases of a febrile character are contagious, is a prelude to the expediency of the isolating of the sick.—*N. O. Med. & Sur. Journal.*

